# Long Noncoding RNA GAS5 Promotes Osteogenic Differentiation of Human Periodontal Ligament Stem Cells by Regulating GDF5 and p38/JNK Signaling Pathway

**DOI:** 10.3389/fphar.2020.00701

**Published:** 2020-05-20

**Authors:** Qiaolin Yang, Yineng Han, Peng Liu, Yiping Huang, Xiaobei Li, Lingfei Jia, Yunfei Zheng, Weiran Li

**Affiliations:** ^1^ Department of Orthodontics, Peking University School and Hospital of Stomatology, Beijing, China; ^2^ Department of Periodontology, Peking University School and Hospital of Stomatology, Beijing, China; ^3^ Central Laboratory, Peking University School and Hospital of Stomatology, Beijing, China

**Keywords:** human periodontal ligament stem cells, osteogenic differentiation, long noncoding RNA, growth arrest specific transcript 5, growth differentiation factor 5

## Abstract

Both extracellular matrix (ECM) and stem cells contribute to the formation of bones. Accumulating evidence proved that the growth differentiation factor 5 (GDF5) plays a vital role in ECM osteogenesis regulation; the use of human periodontal ligament stem cells (hPDLSCs) may contribute to alveolar bone regeneration. Moreover, long noncoding RNAs (lncRNA) serves as a regulator in the growing process of cellular organisms including bone formation. Previous efforts has led us to the discovery that the expression of growth arrest specific transcript 5 (GAS5) changed in the osteogenic differentiation of hPDLSCs. Moreover, the expression of GAS5, as it turns out, is correlated to GDF5. This study attempts to investigate the inner workings of GAS5 in its regulation of osteoblastic differentiation of hPDLSCs. Cell transfection, Alkaline phosphatase (ALP) staining, Alizarin red S (ARS) staining, qRT-PCR, immunofluorescence staining analysis and western blotting were employed in this study. It came to our notice that GAS5 and GDF5 expression increased during osteogenesis induction of hPDLSCs. Knocking down of GAS5 inhibited the osteogenic differentiation of hPDLSCs, whereas overexpressing GAS5 promoted these effects. Molecular mechanism study further demonstrated that overexpressing GAS5 bolsters GDF5 expression and boosts the phosphorylation of JNK and p38 in hPDLSCs, with opposite effects in GAS5 knockdown group. To sum up, long noncoding RNA GAS5 serves to regulate the osteogenic differentiation of PDLSCs *via* GDF5 and p38/JNK signaling pathway. Our findings expand the theoretical understanding of the osteogenesis mechanism in hPDLSCs, providing new insights into the treatment of bone defects.

## Introduction

Alveolar bone loss has been posing perplexing challenges in the field of oral disease for decades. Scholars are committed in the search for more effective methods for bone regeneration. Bone is composed of calcified matrix—derived from extracellular matrix (ECM) and osteocytes (differentiated from osteoblasts). Extracellular matrix, a complicated complex of collagen, proteoglycans and glycoproteins, provides a specialized microenvironment for the proliferation, differentiation, aging and apoptosis of cells (Watt and Huck, 2013). Moreover, ECM, essential in the bone formation process, forms a microenvironment to regulate bioactivities of osteoblasts, providing signals for osteoblasts *via* diversified passages like Wnt and MAPK signaling pathways (Khatiwala et al., 2009; Lisignoli et al., 2017). MSCs are an important group of multipotent cells that can differentiate into a broad range of cell types including osteoblasts(Pittenger et al., 1999). Equipped with self-renewal and multi-differentiation capacity, MSCs are regarded as essential seeding cells in bone tissue engineering(Quarto et al., 2001; Rastegar et al., 2010).

Growth differentiation factor 5 (GDF5), as part of the bone morphogenic protein (BMP) family, is reported to serve an significant function in the tissue differentiation as well as repair of cartilage and bone (Mikic et al., 2004; Miyamoto et al., 2007). Previous study has demonstrated that implantation of GDF5 into ectopic sites in animal models induces the formation of neotendon/ligament-like tissue (Wolfman et al., 1997). GDF-5 is fundamental for articular cartilage maintenance by inducing ECM in articular cartilage and α5 integrin expression (Garciadiego-Cazares et al., 2015). Human cartilage ECM modulating proteins increased in response to GDF-5 protein treatment *via* Wnt signaling pathway (Enochson et al., 2014). Studies have fully investigated and proved the important function of GDF5 in the ECM osteogenic process.

Long noncoding RNAs (lncRNAs) refer to those RNAs with a length of more than 200 nucleotides (Esteller, 2011). In recent years, reports have suggested that lncRNAs serve a critical role in the regulation of the cell growth,differentiation and apoptosis (Guttman et al., 2011). Our research team conducted RNA-seq analysis on mRNA and lncRNA transcriptomes of osteogenically differentiated human periodontal ligament stem cells (hPDLSCs), one type of the mesenchymal stem cells derived from periodontal ligament tissue. And we found more than 200 lncRNAs were expressed differentially in the process of osteogenic induction (Zheng et al., 2018). Among them, lncRNA growth arrest specific transcript 5 (GAS5) showed significant change in expression between the undifferentiated and osteogenically differentiated hPDLSCs. Besides, we analyzed the expression pattern of lncRNAs during osteogenic differentiation of hPDLSCs, and formed the global co-expression networks to detect the genes that may participate in the osteoblast differentiation of hPDLSCs. In the network, we found that long noncoding RNA GAS5 showed strong correlation with GDF5 which is vital in osteoblast differentiation. It is interesting to explore whether GAS5 plays an important role in the process of osteogenic differentiation as GDF5 does. Located in chromosome 1q25.1, GAS5 is comprised of 12 exons with a short open reading frame that lack the ability to encode proteins (Schneider et al., 1988). Although GAS5 does not encode proteins, it is highly expressed in many tissues. The expression of GAS5 turns out to be even higher than many genes that encode proteins, which indicates that it may serve a functional role during the lifetime of the cell (Coccia et al., 1992). Besides, GAS5 is reported to participate in multiple stages of biological processes, like cell proliferation, apoptosis or migration (Pickard and Williams, 2016; Ding et al., 2018; Wang and Kong, 2018). Many studies treat GAS5 as a potent tumor suppressor as its deregulated expression has been linked with a legion of cancers (Ma et al., 2016; Xue et al., 2017)

However, researches on the role of GAS5 in osteogenesis of hPDLSCs are scarce. We attempt, therefore, to determine how GAS5 influences osteogenic induction process of hPDLSCs and explore the possible mechanism.

## Materials and Methods

### Cell Cultivation and Induction

Healthy premolars were collected from three patients (16–20 years of age) in oral maxilla-facial surgery department. The periodontal ligament from the middle third of premolars was gently scraped and digested in trypsin (Gibco) for 5 min. The small pieces of tissue were then seeded onto a culture bottle and incubated in a growth medium (GM), which is composed of alpha minimum essential medium enriched with 10% fetal bovine serum (Gibco) and 1% penicillin and streptomycin in the presence of 5% CO_2_ and a temperature of 37°C. The passages 3–6 of PDLSCs were utilized for subsequent experiments. These cells were identified and positive for mesenchymal stem cell markers CD73, CD105, and CD90 (Zheng et al., 2017). For the induction of differentiation in osteocytes, the hPDLSCs was cultured in osteogenic medium (OM), which is composed of GM supplemented with β-glycerophosphate (10 mM), dexamethasone (100 nM) and vitamin C (200 μM). The culture medium was changed every two days. The researchers obtained their ethical approval from the Ethics Committee (PKUSSIRB-201837096).

### Cell Transfection

The siRNA control (si-NC) together with the small interfering RNAs (si-RNAs) against GAS5 (si-GAS5) and GDF5 (si-GDF5) were designed by Gene Pharma company (Shanghai, China). The si-RNA sequences were presented as following: si-GAS5, 5'-CUUGCCUGGACCAGCUUAATT-3'; si-GDF5, 5'-CCCAAGAAGGAUGAACCCATT-3'; si-NC, 5'-UUCUUCGAACGUGUCACGUTT-3'. When the cells have reached 70–80% of confluence, hPDLSCs were transfected by si-NC, si-GAS5 and si-GDF5 separately using Lipofectamine 3000 (Invitrogen) at 100 nM and Opti-MEM every four days following the manufacturer's instructions. Recombinant lentivirus containing full-length GAS5 (GAS5) and the control (NC) was designed by Gene Pharma company (Shanghai, China). The cells were cultivated with medium containing specific lentivirus for 24 h and then exposed to medium containing puromycin (10 ng/ml) for cell selection.

### Alkaline Phosphatase (ALP) Staining

Following the seven-day induction of osteogenesis, fixing was conducted to the cells using 4% paraformaldehyde for 10 min. Distilled water was then used for washing. Then an NBT/BCIP kit for staining (Co Win Biotech, Beijing, China) was used to conduct the ALP staining according to the protocol.

### Alizarin Red S (ARS) Staining

On the fourteenth day following induction of osteogenesis, fixing of the cells was done in 4% paraformaldehyde for a duration of 10 min. Washing was done thrice using distilled water. After that, 1% Alizarin red S (Sigma-Aldrich St. Louis, MO) staining solution performed staining of the cells for 20 min to assess calcium deposition.

### Quantitative Real-Time Polymerase Chain Reaction (qRT-PCR)

TRIzol reagent (Invitrogen) was used to extract the total RNA from the PDLSCs as directed by the manufacturer's guidelines. The cDNA was then reverse transcribed by utilizing PrimeScriptTM RT Reagent Kit (Takara). The qRT-PCR was then done with the primers listed as follows: GAS5 forward primer: GTGTGGCTCTGGATAGCAC and reverse primer: ACCCAAGCAAGTCATCCATG; RUNX2 forward primer: ACTACCAGCCACCGAGACCA and reverse primer: ACTGCTTGCAGCCTTAAATGACTCT; ALP forward primer: GAACGTGGTCACCTCCATCCT and reverse primer: TCTCGTGGTCACAATGC; OCN forward primer: CACTCCTCGCCCTATTGGCGTG and reverse primer: CCCTCCTGCTTGGACACAAAGA; GDF forward primer: GCTGGGAGGTGTTCGACATC and reverse primer: CACGGTCTTATCGTCCTGGC. Glyceraldehyde 3-phosphate dehydrogenase (GAPDH) was used as normalization and the 2–ΔΔCT method was used for calculations.

### Western Blot Analysis

Collection and lysis of PDLSCs was conducted using RIPA Lysis Buffer which contains 1% protease inhibitor cocktail (Solarbio). Protein concentration was determined by a BCA kit (Thermo) and a total of 30 μg of protein was used for western blot analysis. The primary antibodies against RUNX2 (CST, #12556), GDF5(Abcam, RRID: ab93855), p38 MAPK(Affinity, Cat#AF6456), phosphorylated p38 MAPK (Affinity, Cat#AF4001), JNK(Affinity, Cat#AF6318), phosphorylated JNK(Affinity, Cat#AF3318), ERK(Affinity, Cat#AF0155), phosphorylated ERK(Affinity, Cat#AF1015), and β-ACTIN (Abcam, RRID: ab8226) diluted at 1:1,000 overnight at 4°C. Three washes were done using TBST. Afterwards, incubation of the membranes was done with the anti-rabbit and anti-mouse secondary antibodies (ZB-2301 and ZB-2305, Zhongshan Golden Bridge Biotechnology, Beijing, China) which is diluted at 1:10,000 at room temperature for 1 h. Visualization of the bands was done by enhanced chemiluminescence using the Bio-Rad system for detection (ChemiDocTM MP Imaging System, USA). Intensity of the bands was measured using ImageJ. β-ACTIN internal control was used to ensure equal protein loading.

### Immunofluorescence Staining

Cell plating was done onto sterile glass coverslips and cultured in GM or OM for seven days. Four percent paraformaldehyde was utilized to fix the cells for 20 min at room temperature. The cells were washed (0.01 M PBS) and permeabilized (1% Triton X-100), and then they were blocked with 5% goat serum (ZLI-9022, Zhongshan Golden Bridge Biotechnology, Beijing, China) for 1 h. Primary antibody OCN (Abcam, RRID: ab13418) and anti-rabbit secondary antibody (ZF-0511, Zhongshan Golden Bridge Biotechnology, Beijing, China) were used. DAPI staining was performed to stain nuclei and then the cells were observed and photographed using a confocal system for imaging (LSM 5 EXCITER, Carl Zeiss, Jena, Germany).

### RNA Sequencing

The total RNA was extracted from GAS5 overexpressing and control group using TRIzol reagent (Invitrogen). cDNA libraries were constructed and samples were paired-end sequenced with an Illumina HiSeq 2000 platform. Whole transcriptome sequencing data were mapped to the human genome (hg38) using TopHat2. We used HTseq to count the genes and calculate the reads per kilobase transcriptome per million mapped reads (RPKM) to evaluate the gene expression level. Differentially expressed genes (DEGs) were defined based on fold changes greater than or equal to 2.0 and a false discovery rate of less than 0.05.

### Statistical Analysis

All of the statistical analyses in this study were performed using SPSS 20.0 software (SPSS, Inc., Chicago, IL). Three repeated experiments were done. Results were presented as mean ± SD and analyzed employing Student's t test and one-way analysis of the variance.

## Results

### The Expression of LncRNA GAS5 and GDF5 Is Upregulated During Osteoblast Differentiation of hPDLSCs

**Figure 1:**
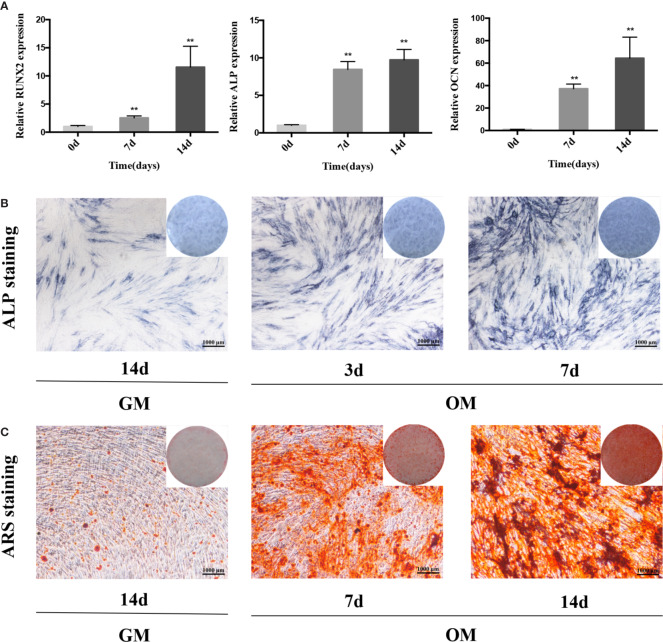
The osteogenic induction of hPDLSCs. **(A)**: The relative expression of osteogenic makers namely RUNX2, ALP and OCN was determined by qRT-PCR during the osteogenesis of hPDLSCs for 0, 7 and 14 days. RNA expression at the above-mentioned time period was normalized to day 0. GAPDH was used as an internal control. **(B)**: The images of ALP staining in hPDLSCs cultivated in GM or OM for 3 and 7 days. **(C)**: The images of ARS staining in hPDLSCs cultivated in GM or OM for 7 and 14 days. **p < 0.01. hPDLSCs, human periodontal ligament stem cells; GAPDH, glyceraldehyde 3-phosphate dehydrogenase; RUNX2, runt-related transcription factor 2; ALP, alkaline phosphatase; OCN, osteocalcin; qRT-PCR, quantitative reverse-transcription polymerase chain reaction; ARS, Alizarin red S; GM, growth medium; OM, osteogenic medium.

**Figure 2:**
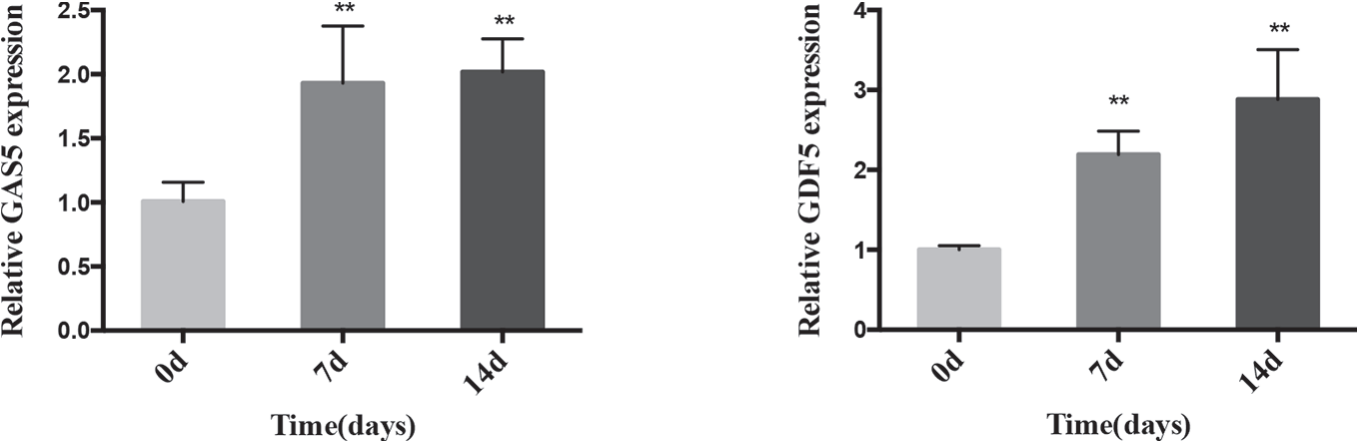
Expression pattern of GAS5 and GDF5 during the osteoblast differentiation of hPDLSCs. The relative expression of GAS5 and GDF5 was determined by qRT-PCR during the osteogenesis of hPDLSCs for 0, 7 and 14 days. RNA expression at the above-mentioned time period was normalized to day 0. GAPDH was used as an internal control. **p <0.01. GAS5, lncRNA growth arrest specific transcript 5; GDF5, growth differentiation factor 5; hPDLSCs, human periodontal ligament stem cells; GAPDH, glyceraldehyde 3-phosphate dehydrogenase; qRT-PCR, quantitative reverse-transcription polymerase chain reaction.

We cultured PDLSCs in OM for 0, 3, 7, and 14 days respectively to prove the effect of osteogenic induction. Quantitative RT-PCR results demonstrated that during the osteogenic differentiation of hPDLSCs the mRNA expression of RUNX2, ALP and OCN (osteogenic markers) significantly increased (**Figure 1A**). The intensity of ALP staining was gradually enhanced at days 0, 3, and 7 of osteogenic differentiation of hPDLSCs (**Figure 1B**). The ARS staining was also deepened progressively at days 0, 7, and 14 of osteogenic differentiation of hPDLSCs (**Figure 1C**). The results indicated that the osteogenic induction was successful. To determine how GAS5 and GDF5 influences the osteogenic differentiation of PDLSCs, we conducted examination of its expression pattern, and found that the expression of GAS5 and GDF5 exhibited a gradual upregulation during the osteogenic differentiation for 14 days (**Figure 2**).

### GAS5 Enhances Osteoblast Differentiation of hPDLSCs

**Figure 3:**
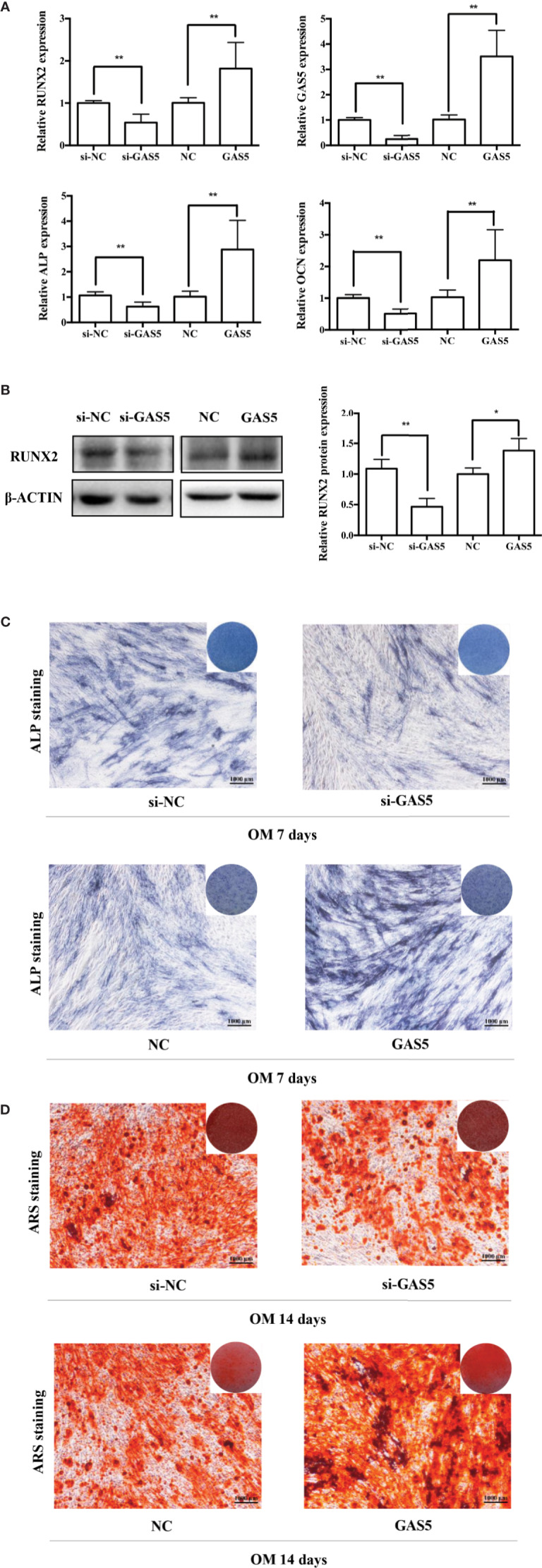
GAS5 enhanced the osteoblast differentiation of hPDLSCs. **(A)**: The efficiency of transient transduction of si-GAS5 and lentivirus infection is measured by qRT-PCR. The mRNA expression of RUNX2, ALP, OCN was measured in si-NC, si-GAS5, NC and GAS5 group on the second day of osteogenic induction. GAPDH mRNA levels were employed for the process of normalization. **(B)**: Western blot analysis of the protein expression of RUNX2 and the internal control β-ACTIN on the third day of osteogenic induction. β-ACTIN was utilized for the normalization relative to si-NC groups. **(C)**: The images of ALP staining in si-NC, si-GAS5, NC and GAS5 group. Cells were cultured in GM or OM for 7 days. **(D)**: Images of ARS staining that stains for mineralized matrix in the si-NC, si-GAS5, NC and GAS5 group were also cultured in GM or OM for 14 days. *p < 0.05, **p < 0.01. hPDLSCs, human periodontal ligament stem cells; GDF5, growth differentiation factor 5; si-NC, small interfering RNA negative control; si-GAS5, the small interfering RNAs that target GAS5; NC, negative control; GAPDH, glyceraldehyde 3-phosphate dehydrogenase; RUNX2, runt-related transcription factor 2; ALP, alkaline phosphatase; OCN, osteocalcin; GM, growth medium; OM, osteogenic medium; qRT-PCR, quantitative reverse-transcription polymerase chain reaction; ARS, Alizarin red S.

**Figure 4:**
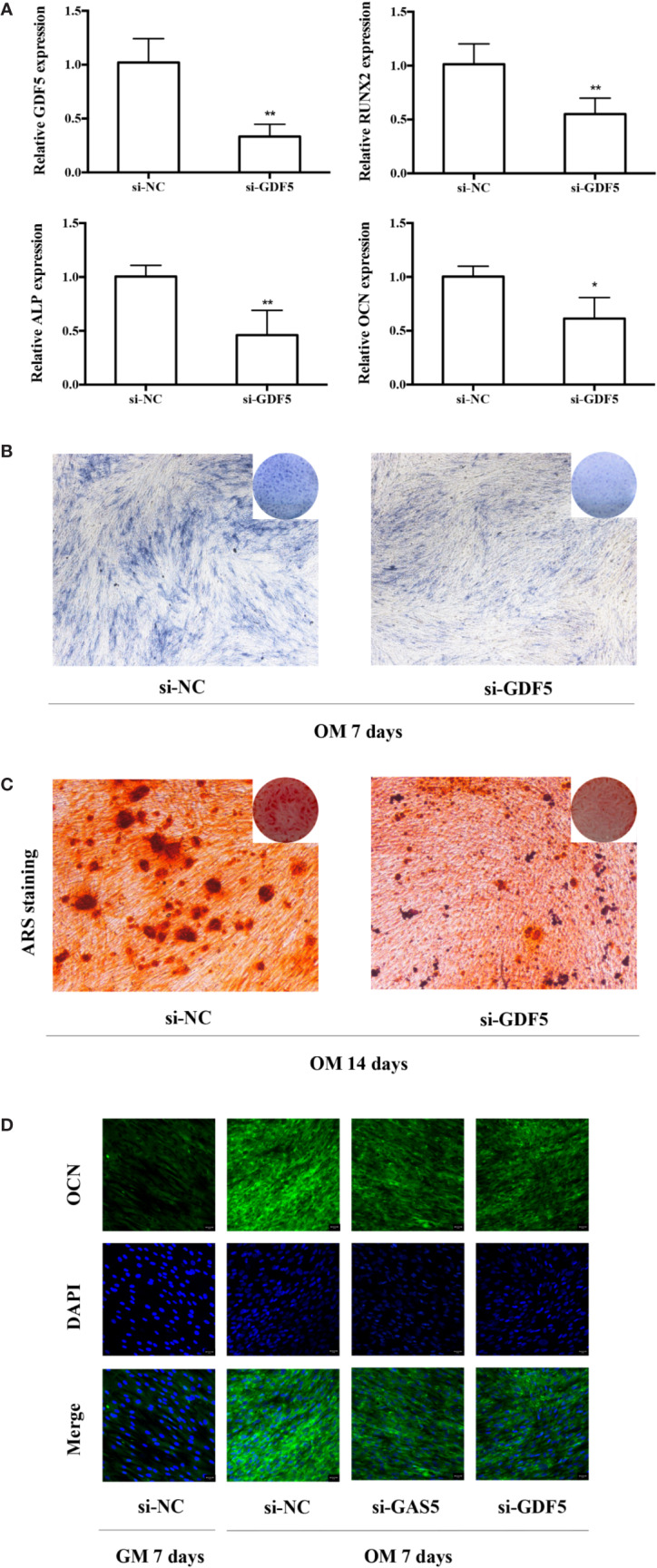
Knockdown of GDF5 inhibits osteoblast differentiation of hPDLSCs. **(A)**: The efficiency of transient transduction of si-GDF5 is measured by qRT-PCR. The mRNA expression of RUNX2, ALP, OCN was measured in si-NC and si-GDF5 group on the second day of osteogenic induction. GAPDH was used for the normalization process relative to si-NC groups. **(B)**: Images of ALP in the si-GDF5 and si-NC groups. Cells were cultured in OM for 7 days. **(C)**: Images of ARS which stains for mineralized matrix in the si-GDF5 and si-NC groups. Cells were cultured in OM for 14 days. **(D)**: Immunofluorescence staining analysis of OCN protein expression at si-NC, si-GAS5 and si-GDF5 groups. Cells were cultured in GM or OM for 7 days. *p < 0.05, **p < 0.01. hPDLSCs, human periodontal ligament stem cells; si-NC, small interfering RNA negative control; si-GDF5, the small interfering RNAs that target GDF5; GAPDH, glyceraldehyde 3-phosphate dehydrogenase; RUNX2, runt-related transcription factor 2; ALP, alkaline phosphatase; OCN, osteocalcin; GM, growth medium; OM, osteogenic medium; qRT-PCR, quantitative reverse-transcription polymerase chain reaction; ARS, Alizarin red S.

To investigate the function of GAS5, we used si-GAS5 to knock down and lentivirus to overexpress GAS5 in hPDLSCs. The qRT-PCR results indicated that the expression of GAS5 declined nearly 80% using si-GAS5, and the efficiency of lentivirus transfection showed a more than 4-fold increase in comparison with the control group. Knocking down GAS5 caused a reduction in the mRNA levels of RUNX2, ALP and OCN compared to si-NC group, whereas overexpression of GAS5 upregulated those osteogenic related genes (**Figure 3A**). Western blot analysis further indicated that silencing GAS5 inhibited the expression of osteogenic related proteins RUNX2 and overexpressing GAS5 caused an increase in RUNX2 protein expression (**Figure 3B**). The staining of ALP decreased after seven-day osteogenic induction of GAS5 knockdown PDLSCs, whereas significantly enhanced in the GAS5 overexpression group in comparison with NC group (**Figure 3C**). Consistently, after the 14-day OM induction, matrix mineralization was inhibited in the GAS5 knockdown group and was promoted in the GAS5 overexpression group as revealed by ARS staining (**Figure 3D**). Besides, analysis by immunofluorescence revealed downregulated protein level of OCN in PDLSCs with si-GAS5 compared to that with si-NC at day 7 of osteogenic differentiation (**Figure 4D**).

### Downregulation of GDF5 Inhibits Osteoblast Differentiation of hPDLSCs

To help elucidate the role of GDF5 in osteogenic differentiation of hPDLSCs, similarly, we used si-GDF5 to realize the knockdown of GDF5 and the expression of GDF5 was reduced by 70–80%. Several osteogenic markers (RUNX2, ALP, OCN) was downregulated after knocking down GDF5 as tested by qRT-PCR (**Figure 4A**). The intensity of ALP staining decreased in the GDF5 knockdown group after the 7-day osteogenic differentiation (**Figure 4B**), and the ARS staining weakened consistently after the 14-day osteogenic differentiation (**Figure 4C**), indicating that downregulation of GDF5 inhibits osteoblast differentiation of hPDLSCs. In addition, immunofluorescence staining analysis further demonstrated that the negative effect of knocking down GDF5 on osteogenic differentiation of hPDLSCs (**Figure 4D**).

### GAS5 Presents a Co-Expression Relationship With GDF5

**Figure 5:**
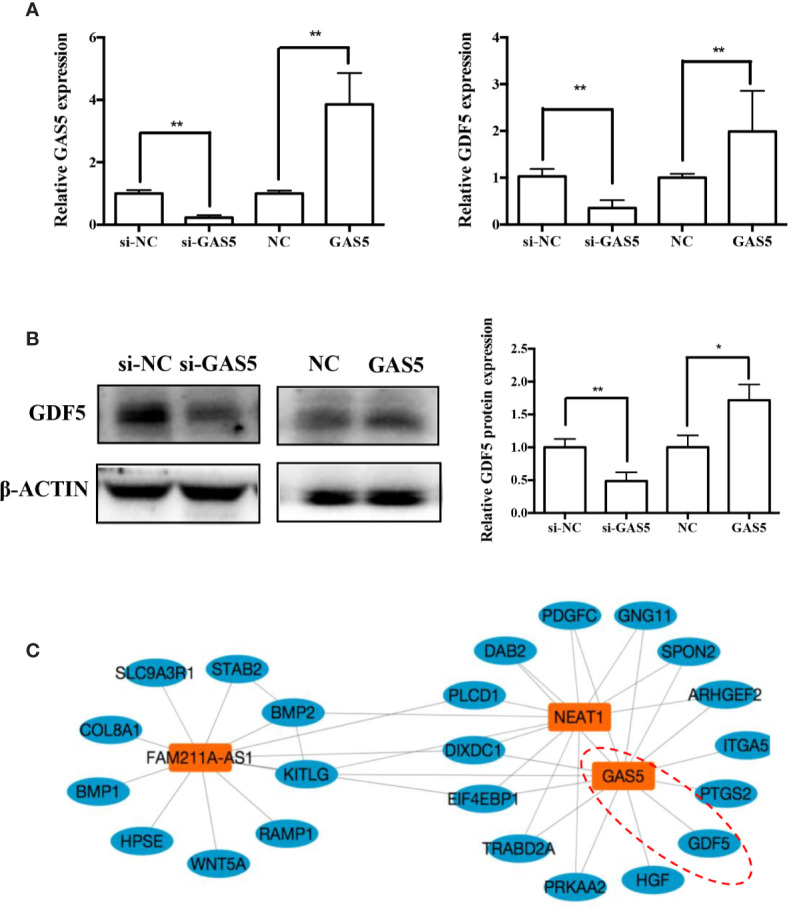
GAS5 regulated the expression of GDF5. **(A)**: The mRNA expression of GDF5 as is detected by qRT-PCR in si-NC, si-GAS5, NC and GAS5 group. GAPDH was employed for the normalization. **(B)**: Western blot analysis of the protein expression of GDF5 and the internal control β-ACTIN in si-NC, si-GAS5, NC and GAS5 group. The histogram demonstrates the quantification of the band intensities. **(C)**: The co-expression network connections of the module, containing GAS5 and NEAT1. *p < 0.05, **p < 0.01. GAS5, lncRNA growth arrest specific transcript 5; GDF5, growth differentiation factor 5; si-NC, small interfering RNA negative control; si-GAS5, the small interfering RNAs that target GAS5; NC, negative control; GAPDH, glyceraldehyde 3-phosphate dehydrogenase; qRT-PCR, quantitative reverse-transcription polymerase chain reaction.

In previous research, we analyzed the expression pattern of lncRNA during osteogenic differentiation of hPDLSCs, and visualized the global co-expression networks to find out the genes that may participate in the osteoblast differentiation of hPDLSCs. In the network, we found that GAS5 showed strong correlation with GDF5 that were found to play a vital role in osteoblast differentiation (**Figure 5A**). To confirm whether GDF5 is influenced by GAS5, we used RNA sequencing (RNA-seq) to identify differentially expressed genes in GAS5-overexpression PDLSCs compared to the control group (NC). And we investigated the potential regulatory roles played by differentially expressed genes *via* Gene Ontology (GO) and Kyoto Encyclopedia of Genes and Genomes (KEGG) pathway analyses. Finally, we found 509 genes downregulated and 156 genes upregulated in the GAS5 overexpression group. Among these, the expression of GDF5 was increased to nearly 4-fold. The qRT-PCR results were consistent with the RNA-seq data. The mRNA expression of GDF5 was decreased after knocking down GAS5 and upregulated by overexpressing GAS5 (**Figure 5B**). Furthermore, western blot analysis demonstrated that GDF5 was decreased in si-GAS5 group and upregulated in the GAS5 overexpressing group (**Figure 5C**).

### GAS5 Promotes and Knocking Down GDF5 Inhibits Osteogenic Differentiation of hPDLSCs Partly *via* Alleviating p38/JNK Phosphorylation

**Figure 6:**
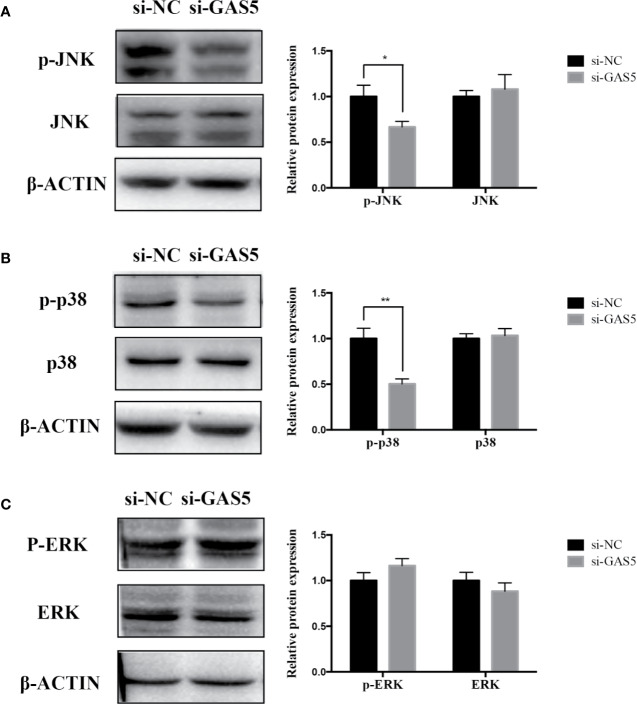
Knockdown of GAS5 weakened the phosphorylation of JNK and p38 in hPDLSCs. **(A)**: Western blot analysis of phosphorylated JNK (p-JNK), JNK and β-ACTIN in hPDLSCs transfected with si-NC or si-GAS5. Histogram showed the quantification of the band intensities. **(B)**: Western blot analysis of phosphorylated p38 (p-p38), p38 mitogen-activated protein kinase (p38), and β-ACTIN in hPDLSCs transfected with si-NC or si-GAS5. Histogram showed the quantification of the band intensities. **(C)**: Western blot analysis of phosphorylated ERK (p-ERK), ERK and β-ACTIN in hPDLSCs transfected with si-NC or si-GAS5. Histogram showed the quantification of the band intensities. *p < 0.05, **p < 0.01. hPDLSCs, human periodontal ligament stem cells; GAS5, lncRNA growth arrest specific transcript 5; si-NC: small interfering RNA negative control; si-GAS5: the small interfering RNAs that target GAS5; NC, negative control; JNK, c-Jun N-terminal kinase; ERK, intracellular signal-regulated kinase 1/2.

**Figure 7:**
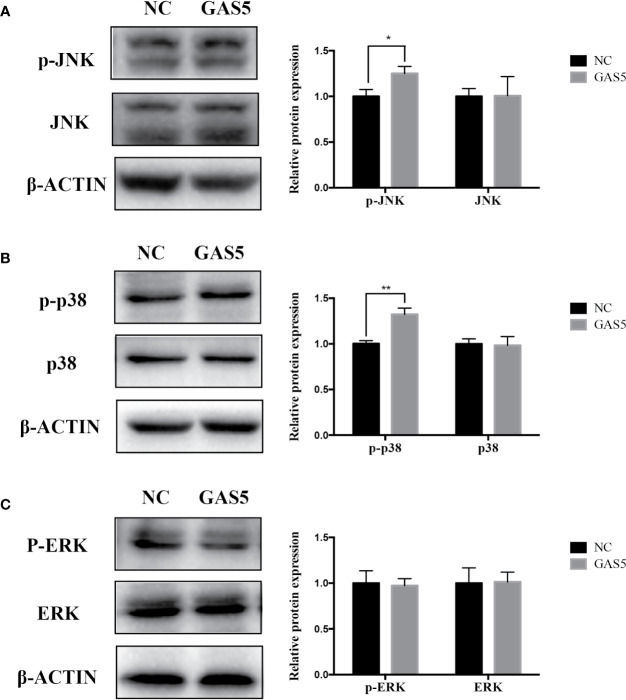
Overexpressing GAS5 enhanced the phosphorylation of JNK and p38 in hPDLSCs. **(A)**: Western blot analysis of phosphorylated JNK (p-JNK), JNK and β-ACTIN in hPDLSCs transfected with NC or GAS5. Histogram showed the quantification of the band intensities. **(B)**: Western blot analysis of phosphorylated p38 (p-p38), p38 mitogen-activated protein kinase (p38), and β-ACTIN in hPDLSCs transfected with NC or GAS5. Histogram showed the quantification of the band intensities. **(C)**: Western blot analysis of phosphorylated ERK (p-ERK), ERK and β-ACTIN in hPDLSCs transfected with NC or GAS5. Histogram showed the quantification of the band intensities. *p < 0.05, **p < 0.01. hPDLSCs, human periodontal ligament stem cells; GAS5, lncRNA growth arrest specific transcript 5; NC, negative control; JNK, c-Jun N-terminal kinase; ERK, intracellular signal-regulated kinase 1/2.

**Figure 8:**
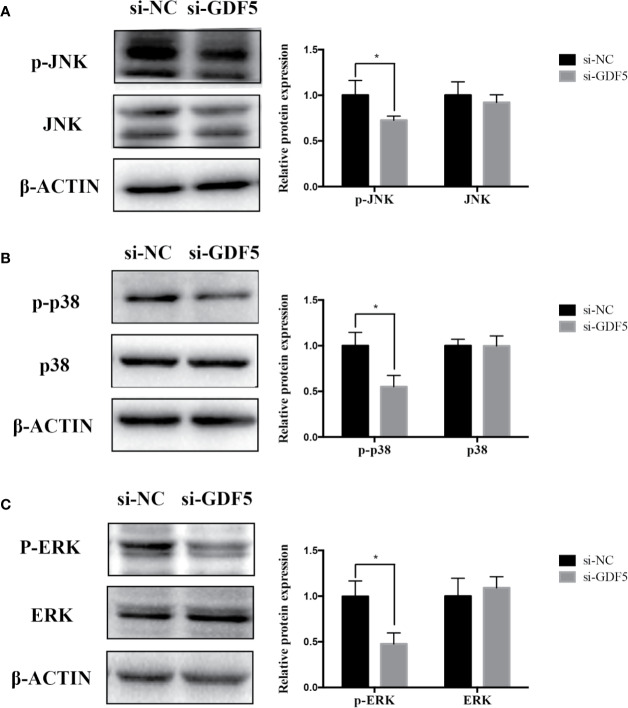
Knockdown of GDF5 weakened the phosphorylation of JNK and p38 in hPDLSCs. **(A)**: Western blot analysis of phosphorylated JNK (p-JNK), JNK and β-ACTIN in hPDLSCs transfected with si-NC or si-GDF5. Histogram showed the quantification of the band intensities. **(B)**: Western blot analyses of phosphorylated p38 (p-p38), p38 mitogen-activated protein kinase (p38), and β-ACTIN in hPDLSCs transfected with si-NC or si-GDF5. Histogram showed the quantification of the band intensities. **(C)**: Western blot analyses of phosphorylated ERK (p-ERK), ERK and β-ACTIN in hPDLSCs transfected with si-NC or si-GDF5. Histogram showed the quantification of the band intensities. *p <0.05. hPDLSCs, human periodontal ligament stem cells; GDF5: growth differentiation factor 5; si-NC: small interfering RNA negative control; si-GDF5: the small interfering RNAs that target GDF5; NC, negative control; JNK, c-Jun N-terminal kinase; ERK, intracellular signal-regulated kinase 1/2.

The relevant studies showed that p38 MAPK signaling pathway serve a significant role in the bone formation and inflammation, and it was activated by the TGF-β superfamily of proteins, including BMPs (Li et al., 2014). Furthermore, our group have confirmed that GDF5 can regulate osteogenic differentiation partially *via* phosphorylation of p38 and SMAD1/5/8 (Li X. et al., 2018). Thus, we investigated whether GAS5 and GDF5 can regulate the proteins in MAPK signaling pathway. To evaluate the levels of p38, phosphorylated p38 (p-p38), extracellular signal-regulated kinase 1/2 (ERK), phosphorylated ERK (p-ERK), c-Jun N-terminal kinase (JNK) and phosphorylated JNK (p-JNK) in MAPK pathway in the si-GAS5, si-GDF5, si-NC,GAS5 and NC treated hPDLSCs, western blot analysis was conducted. The results showed that the phosphorylation of p38 and JNK decreased in si-GAS5 group and there was no significant difference in the phosphorylation of ERK (**Figure 6**). The phosphorylation of p38 and JNK increased in GAS5 overexpressing group and there was no significant difference in the phosphorylation of ERK (**Figure 7**). The phosphorylation of p38, JNK and ERK all declined in si-GDF group (**Figure 8**). 

## Discussion

GAS5 was overexpressed in growth arrest cells and was thus named growth arrest-specific 5 (Schneider et al., 1988). Ever since the discovery, researches related to GAS5 has been increasing dramatically. Its expression has been meticulously recorded in a large scope of tissues and its function has been thoroughly studied over different stages of development (Pickard and Williams, 2015). Its role as a tumor suppressor lncRNA attracted much attention when its low-expression was detected in cancers such as non-small cell lung cancer and colorectal carcinoma (Yang et al., 2017; Ding et al., 2018). Studies showed that GAS5 is essential in modulating the pluripotency and self-renew ability of mouse embryonic stem cells. Besides, it represses endodermal lineage differentiation and promotes induced pluripotent stem cells reprogramming (Tu et al., 2018). Also, its involvement in inflammation was studied with inconsistent results (Sun et al., 2017; Li F. et al., 2018). The function of GAS5 in osteogenesis, however, seems to be neglected by academia. To explore the uncharted waters, i.e. its role in osteogenic differentiation, we detected the expression pattern of GAS5 in osteogenic induction of hPDLSCs, and used si-GAS5 and GAS5 to confirm its regulatory function in mRNAs and proteins related to bone formation. We used RNA-seq to explore the co-expression network connections and found the relationship between GAS5 and GDF5, following that we found that GAS5 can promote osteogenesis of hPDLSCs by upregulating GDF5 *via* a p38/JNK signaling pathway.

Through complicated regular mechanisms, lncRNAs play an essential part throughout regulatory processes of genes. lncRNAs can mediate epigenetic regulation *via* binding to proteins, such as chromatin regulatory complexes before the transcription processes (Wutz and Gribnau, 2007). During the transcription process, lncRNAs can interact with transcriptional factors and affect its activity (Feng et al., 2006). Besides, the post-transcriptional processes is also influenced by lncRNAs, mostly through competing endogenous RNA (ceRNA) mechanism (Poliseno et al., 2010). As for GAS5, the specific regulatory mechanism remains unclear. It is reported that GAS5 can specifically unite with DNA segments in the glucocorticoid receptor (GR), inhibiting the bonding between the GR and glucocorticoid response elements in target genes (Kino et al., 2010). Besides, GAS5 can integrate with eukaryotic translation initiation factor-4E or act as competing endogenous RNA (ceRNA), such as miR-21 (Hu et al., 2014; Song et al., 2014). The relationship between GAS5 and GDF5 is uncovered by the network analysis in our previous work. Then we found that GAS5 can regulate the expression of GDF5 in the osteogenic differentiation of PDLSCs. It is reported that miR-21 promoted the chondrogenesis of osteoarthritis by directly targeting GDF5 (Zhang et al., 2014). We propose that there might be a lncRNA-miRNA regulatory loop between GAS5 and GDF5; miR-21 might potentially serve as one of the links. The specific path in which GAS5 interacts with GDF5, however, remains to be investigated.

Mitogen-activated protein kinases (MAPKs) are a family of evolutionarily conserved serine/threonine kinases that can help to transduce extracellular stimuli into cells and nuclei, participating in multiple biological processes such as cellular proliferation, differentiation, and apoptosis (Bluthgen and Legewie, 2008; Kim and Choi, 2015). MAPKs have three main subfamilies, namely, extracellular signal-regulated kinase (ERK), c jun N-terminal kinase (JNK), and p38. Previous studies have demonstrated that MAPK serves a vital role in the regulation of bone mass *via* control of osteoblast differentiation (Higuchi et al., 2002; Greenblatt et al., 2010). To further investigate the mechanism by which GAS5 promoted the osteogenic differentiation of PDLSCs, we detected the protein levels of JNK, p38, ERK, and their phosphorylated forms. The results indicated that GAS5 promoted the phosphorylated levels of JNK, and p38, whereas not significantly changed the expression levels of ERK.

Studies have indicated that the JNK and p38 phosphorylation is involved in the osteogenesis of PDLSCs. It is demonstrated that cannabinoid receptor 1 enhanced the osteo/dentinogenic differentiation ability of periodontal ligament stem cells *via* p38 MAPK and JNK in an inflammatory environment (Yan et al., 2019). Bone morphogenetic protein-9 induces PDLSCs osteogenic differentiation through the ERK and p38 signal pathways (Ye et al., 2014). In the present study, GAS5 increased the phosphorylated levels of JNK and p38, which indicated GAS5 enhanced osteogenic differentiation of PDLSCs possibly through activation of p38 MAPK and JNK signaling pathway. In addition to the MAPK pathway, other pathway like Wnt/β-catenin, BMP/TGF-β pathways have also been reported to be involved in the osteogenesis of PDLSCs (Cao et al., 2017; Kim et al., 2018). There are intricate cross-talks between the pathways, forming a complex regulatory network; all pathways have not been exhaustively studied. More experiments are expected for a better understanding of the perplexing picture of regulatory network.

Loss of homeostasis in ECM can contribute to cartilage defect and bone disorders, such as osteoarthritis and osteoporosis (Paiva and Granjeiro, 2017; Rahmati et al., 2017). Our findings attempt to offer new perspectives into the ability of GAS5 to promote hPDLSCs osteogenic differentiation partially *via* regulating GDF5 and participating in p38/JNK pathway. Our results shed lights on the application of hPDLSCs combined with GAS5, which could be a potentially useful approach in stimulating osteogenesis in bone tissue engineering. And its regulatory network may cast some light to the mechanism of bone disorders.

## Data Availability Statement

RNA sequence data has been uploaded into NCBI Sequence Read Archive with accession numbers “SRR11093247” and “SRR11093246”.

## Ethics Statement

The studies involving human participants were reviewed and approved by the Ethics Committee of Peking University School of Stomatology, Beijing, People's Republic of China (PKUSSIRB—2011007). Written informed consent to participate in this study was provided by the participants' legal guardian/next of kin.

## Author Contributions

QY contributed to cell experiment, collection of data and analysis, statistical analysis, and writing of the manuscript. YHa contributed to cell experiment. PL, XL, and YHu contributed to the data collection and statistical analysis. WL, YZ, and LJ contributed to the design of this study as well as the revision of the manuscript. All authors have read and approved the final article.

## Funding

This research was financed by the grants from the Beijing Natural Science Foundation (No. 7172239), the National Natural Science Foundation of China (Nos. 81700938; 81670957; 81772876).

## Conflict of Interest

The authors declare that the research was conducted in the absence of any commercial or financial relationships that could be construed as a potential conflict of interest.

## Supplementary Material

Data Sheet 1 Raw data.
