# Supplementary figures and images for: Long Noncoding RNA GAS5 Promotes Osteogenic Differentiation of Human Periodontal Ligament Stem Cells by Regulating GDF5 and p38/JNK Signaling Pathway
Source: Front Pharmacol. 2020 May 20;11:701. doi: 10.3389/fphar.2020.00701 (PMC7251029; doi:10.3389/fphar.2020.00701)

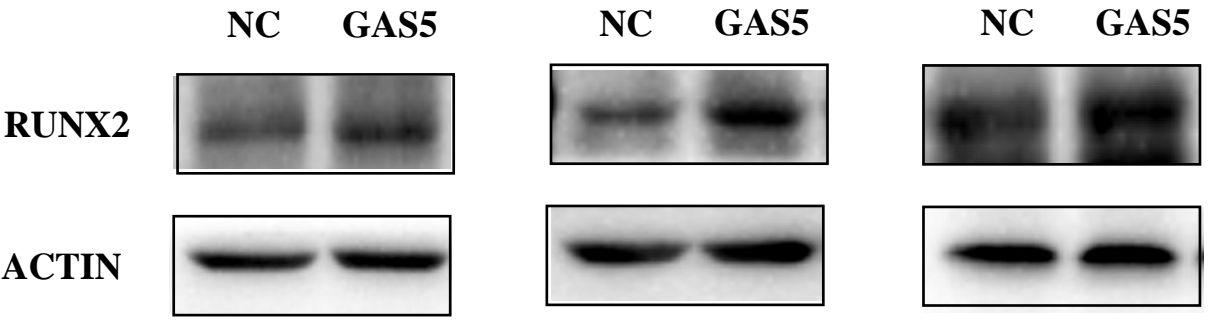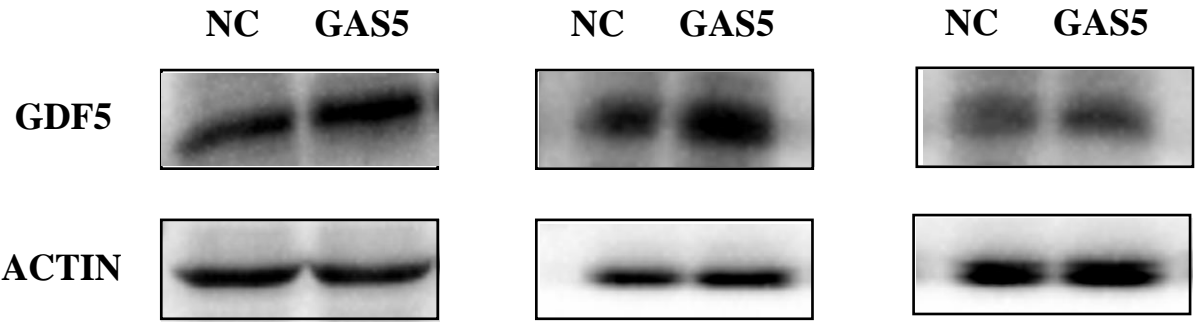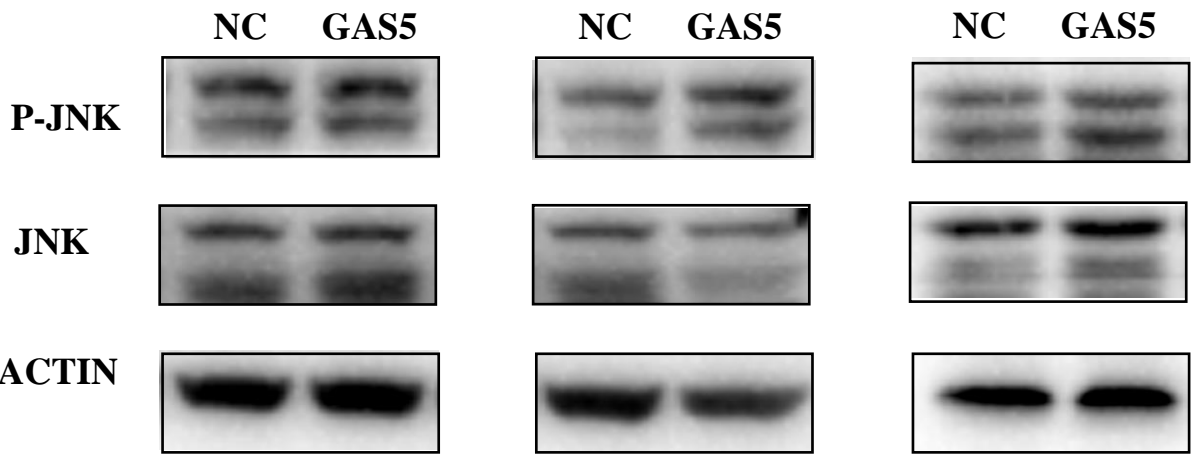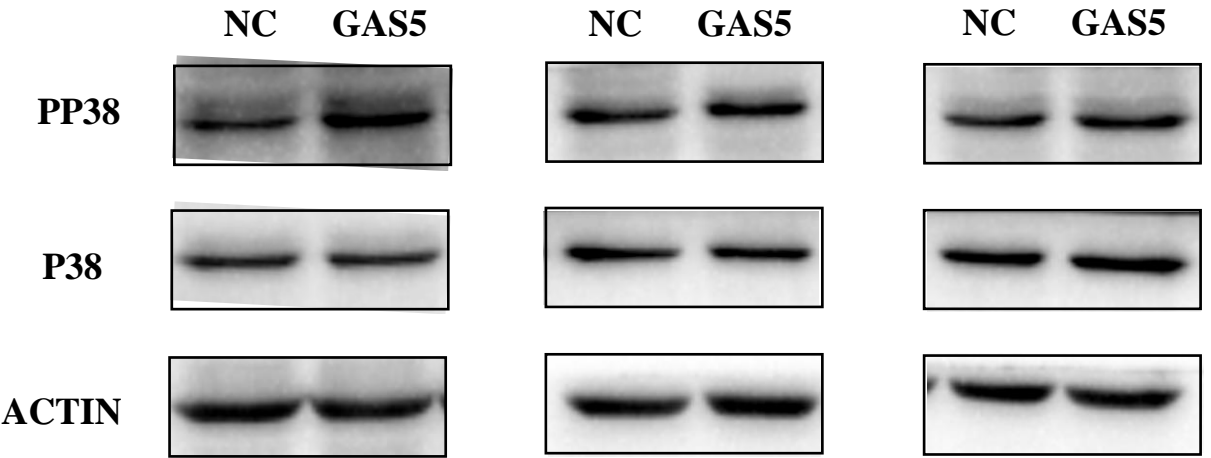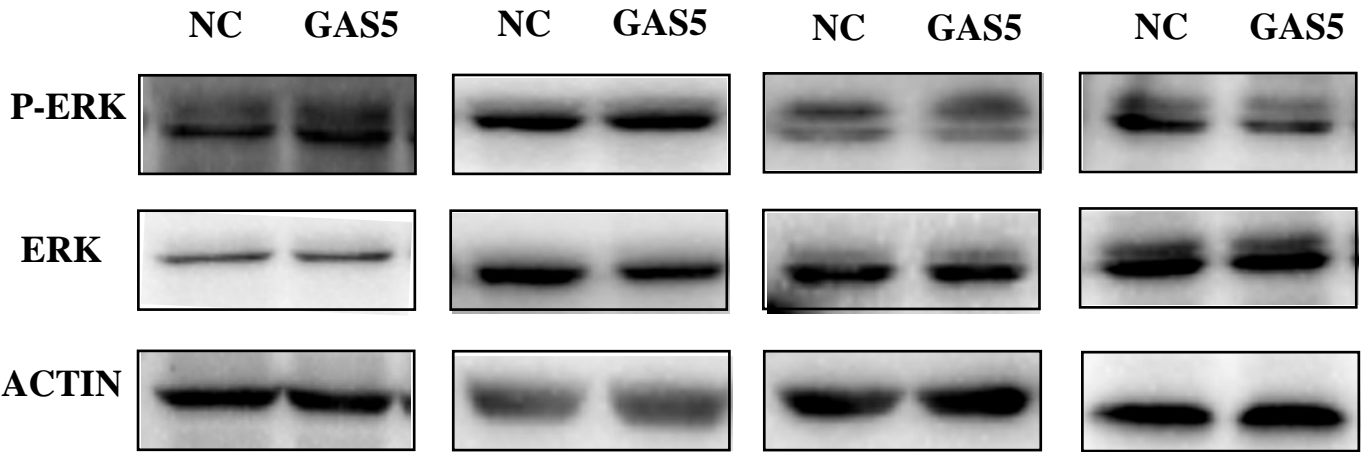

Supplement: Data Sheet 1 — Raw data. [file DataSheet_1.zip › raw data/raw dataí¬western blot/wb╩2╛▌╒√└φ(gas5).pdf]

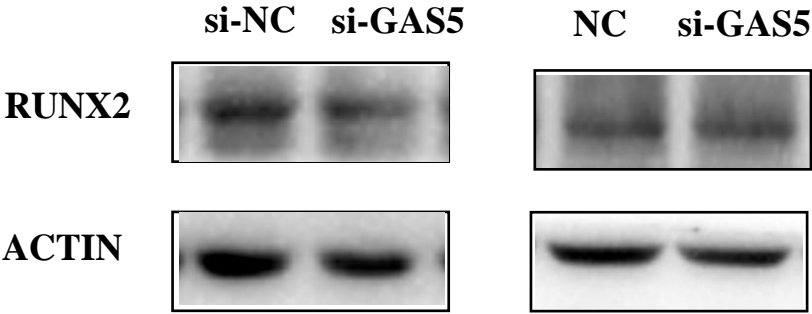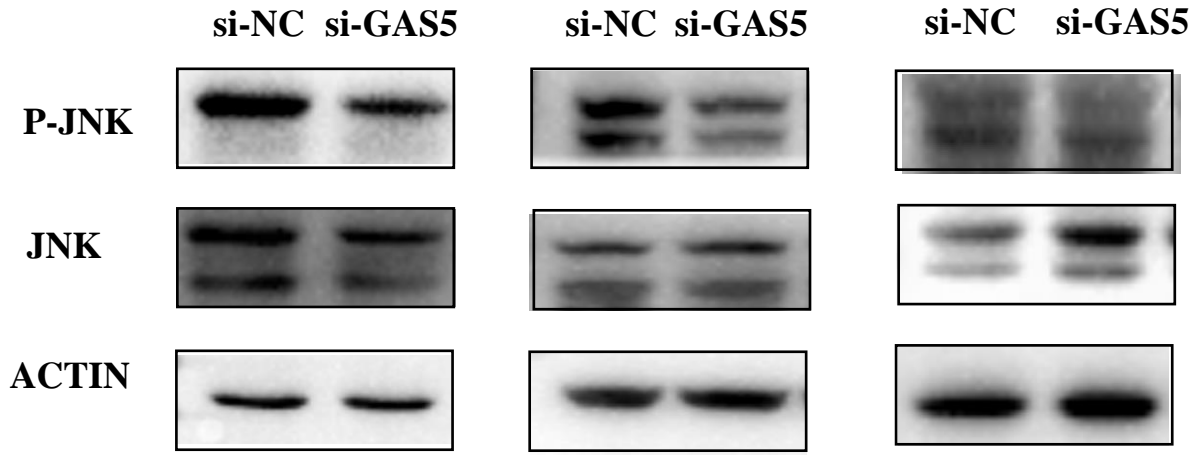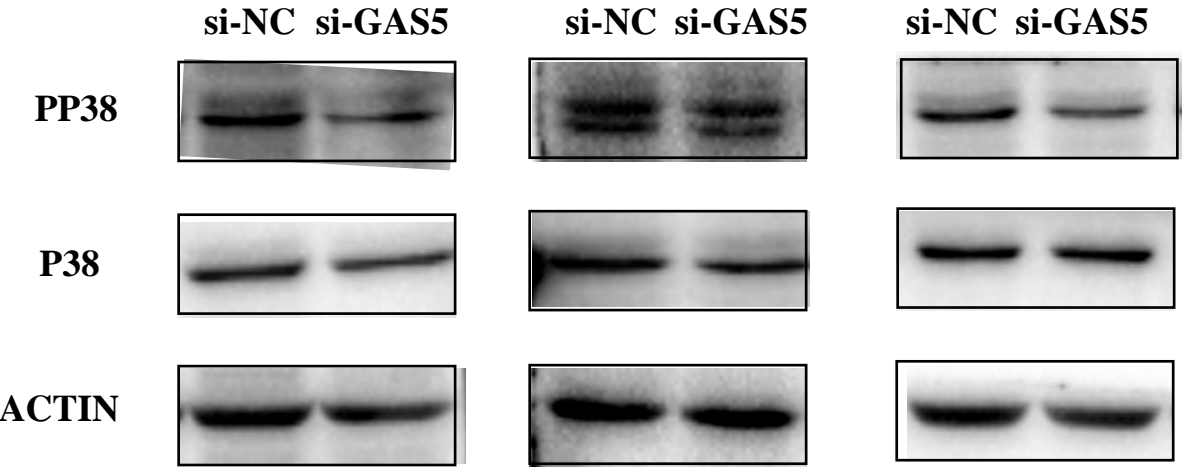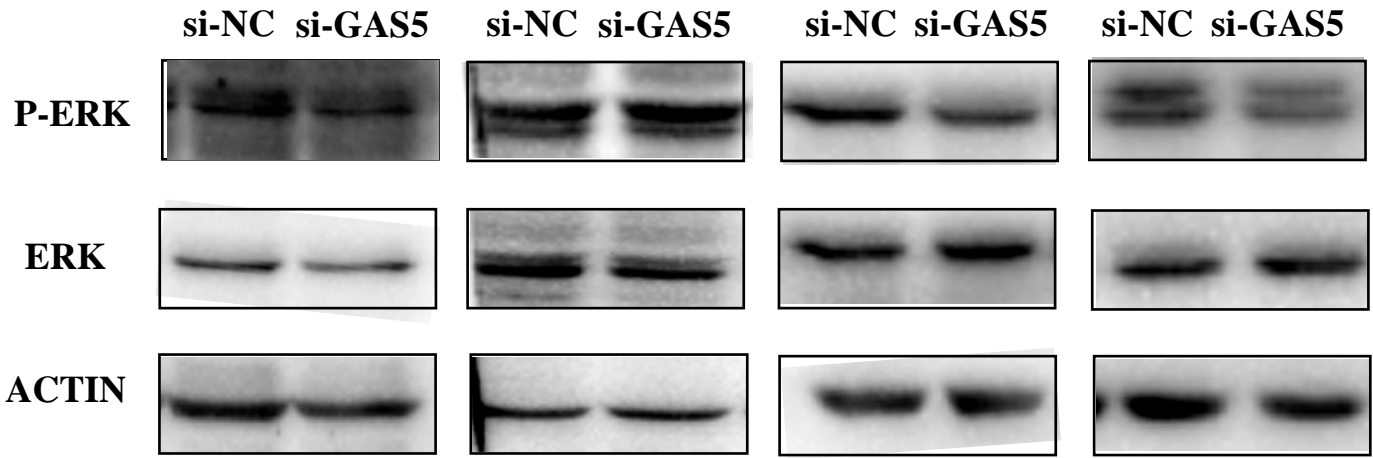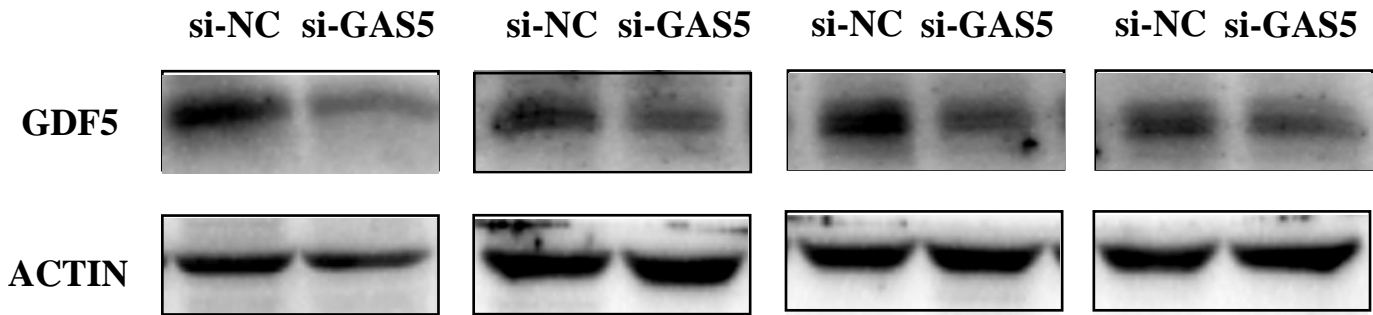

Supplement: Data Sheet 1 — Raw data. [file DataSheet_1.zip › raw data/raw dataí¬western blot/wb╩2╛▌╒√└φ(si-gas5).pdf]

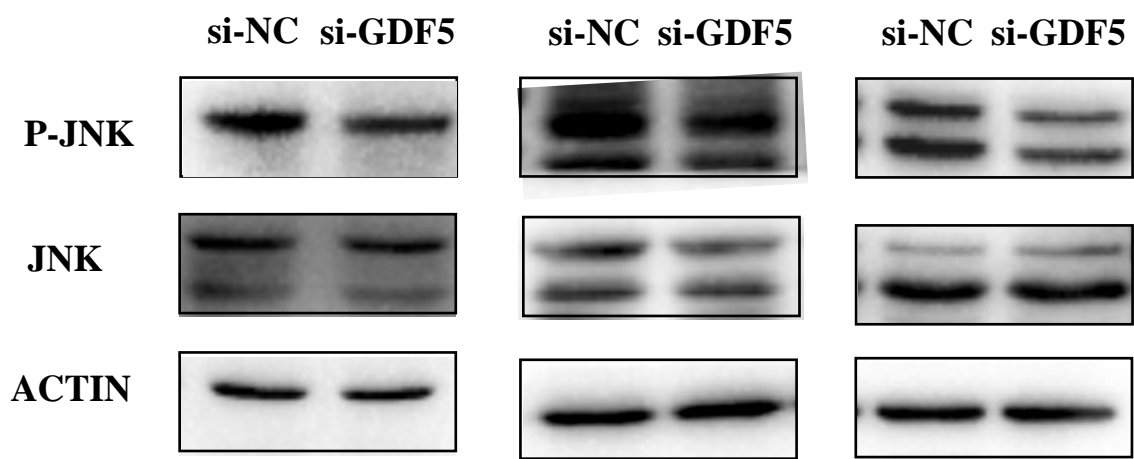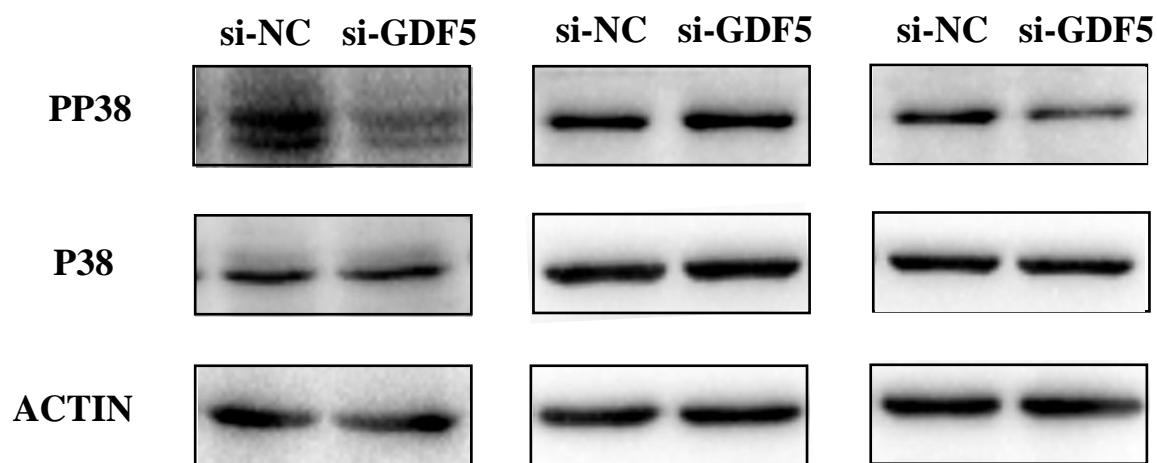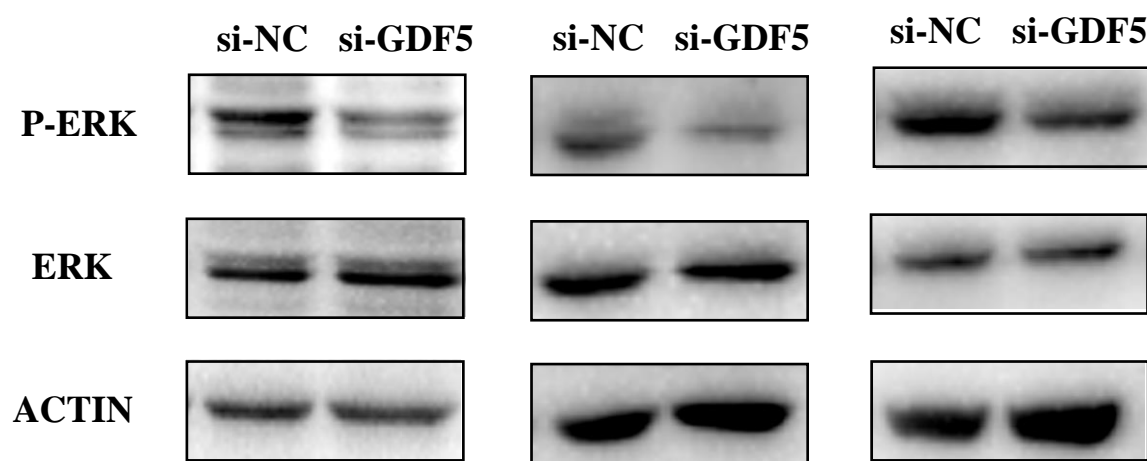

Supplement: Data Sheet 1 — Raw data. [file DataSheet_1.zip › raw data/raw dataí¬western blot/wb╩2╛▌╒√└φ(si-gdf5).pdf]
